# Complete Genome of Citrobacter freundii Siphophage Stevie

**DOI:** 10.1128/genomeA.01434-14

**Published:** 2015-02-05

**Authors:** Justin P. Shaw, Carlos A. Aviles Medina, Yi Chen, Adrian J. Luna, Adriana C. Hernandez, Gabriel F. Kuty Everett

**Affiliations:** a Center for Phage Technology, Texas A&M University, College Station, Texas, USA; b Departamento de Genética y Biología Molecular, Centro de Investigación y Estudios Avanzados del Instituto Politécnico Nacional (CINVESTAV-IPN), México DF, Mexico

## Abstract

Citrobacter freundii is an opportunistic pathogen responsible for many urinary tract infections acquired in hospitals and is thus a concern for public health. C. freundii phage Stevie might prove beneficial as a treatment against these infections. The complete genome of Stevie and its key features are described here.

## GENOME ANNOUNCEMENT

Citrobacter, a Gram-negative bacteria, includes many opportunistic pathogens. C. freundii causes urinary and respiratory tract infections, many of which have been found to be resistant to a number of antibiotics (1, 2). This makes bacteriophages infecting C. freundii, such as siphophage Stevie, described here, of particular interest for combating multi-drug resistance (3).

Bacteriophage Stevie was isolated from a dirt sample collected in College Station, TX. Phage DNA was sequenced in an Illumina MiSeq 250-bp paired-end run with a 550-bp insert library at the Genomic Sequencing and Analysis Facility at the University of Texas (Austin, TX). Quality-controlled trimmed reads were assembled to a single contig at 20.9 fold coverage using Velvet version 1.2.10. Genes were predicted using GeneMarkS (4) and corrected using software tools available on the Center for Phage Technology (CPT) Galaxy instance (https://cpt.tamu.edu/galaxy-public/) (5–7). Morphology was determined using transmission electron microscopy performed at the Texas A&M University Microscopy and Imaging Center.

Stevie is a T1-like phage with a 49,759-bp partially permuted genome, G+C content of 43.7%, 91.5% coding density, and 91 predicted coding sequences. Stevie shares 47.7, 62.8, and 44.2 percent nucleotide sequence identity to siphophages T1 (NC_005833), TLS (NC_009540), and FSL SP-030 (NC_021779), respectively, as determined by Emboss Stretcher (8). It has an abundance of rho-independent terminators, a distinguishing feature of T1-like phages, with 21 predicted in its genome compared to the 17 found in phage T1 (9). Sixteen occurrences of a 22-bp repeat sequence (consensus-VWATAGCAYKWWTTGYTAAAAV) were identified in the sequence of Stevie. A comparable repeat with 20 occurrences was identified in the T1 genome (9). The repeat sequence is asymmetric, predominantly intergenic, and oriented in one direction with respect to the direction of transcription. A 13-bp repeat with similar features was described in mycobacteriophage L5 as a stoperator involved in the maintenance of transcriptional silence of the integrated prophage (10). The role these repeats play in the lytic lifestyle of T1 and Stevie is unknown.

Genes encoding T1-like morphogenesis proteins include the prohead protease, major capsid protein, tail fibers, tail assembly proteins, tape measure protein, and tape measure chaperones (complete with a conserved translational frameshift) (11). Few DNA replication/recombination proteins were identified (single-strand annealing protein, single-strand binding protein, primase, helicase, and an endonuclease). Stevie encodes a DNA (N^6^-adenine)-methyltransferase and presumably methylates its DNA-like phage T1 (12, 13). Stevie uses a headful DNA-packaging mechanism as determined by homology of TerL to large terminase proteins of phages with known *pac*-type strategies (14).

Similar to T1-like phage TLS, Stevie encodes a T4 Stp-like peptide that protects the phage from specific host restriction enzymes (15). In T4, the Stp-mediated restriction enzyme protection activates a host tRNA^Lys^-specific anticodon nuclease (ACNase), the action of which is mitigated by phage encoded polynucleotide kinase, also present in the Stevie genome. The lysis cassette consists of a predicted (class-II) pinholin (6), a R^21^-like signal anchor release (SAR) lysozyme (16, 17), and a unimolecular spanin (18).

### Nucleotide sequence accession number.

The genome sequence of phage Stevie was deposited in GenBank under the accession no. KM236241.

## References

[1] Shih CC, Chen YC, Chang SC, Luh KT, Hsieh WC 1996 Bacteremia due to *Citrobacter* species: significance of primary intraabdominal infection. Clin Infect Dis 23:543–549. doi:10.1093/clinids/23.3.543. 8879778

[2] Kim PW, Harris AD, Roghmann MC, Morris JG Jr, Strinivasan A, Perencevich EN 2003 Epidemiological risk factors for isolation of ceftriaxone-resistant versus -susceptible *Citrobacter freundii* in hospitalized patients. Antimicrob Agents Chemother 47:2882–2887. doi:10.1128/AAC.47.9.2882-2887.2003. 12936989 PMC182594

[3] Abedon ST, Kuhl SJ, Blasdel BG, Kutter EM 2011 Phage treatment of human infections. Bacteriophage 1:66–85. doi:10.4161/bact.1.2.15845. 22334863 PMC3278644

[4] Besemer J, Lomsadze A, Borodovsky M 2001 GeneMarkS: a self-training method for prediction of gene starts in microbial genomes. Implications for finding sequence motifs in regulatory regions. Nucleic Acids Res 29:2607–2618. doi:10.1093/nar/29.12.2607. 11410670 PMC55746

[5] Camacho C, Coulouris G, Avagyan V, Ma N, Papadopoulos J, Bealer K, Madden TL 2009 BLAST+: architecture and applications. BMC Bioinformatics 10:421. doi:10.1186/1471-2105-10-421. 20003500 PMC2803857

[6] Hunter S, Apweiler R, Attwood TK, Bairoch A, Bateman A, Binns D, Bork P, Das U, Daugherty L, Duquenne L, Finn RD, Gough J, Haft D, Hulo N, Kahn D, Kelly E, Laugraud A, Letunic I, Lonsdale D, Lopez R, Madera M, Maslen J, McAnulla C, McDowall J, Mistry J, Mitchell A, Mulder N, Natale D, Orengo C, Quinn AF, Selengut JD, Sigrist CJ, Thimma M, Thomas PD, Valentin F, Wilson D, Wu CH, Yeats C 2009 InterPro: the integrative protein signature database. Nucleic Acids Res 37:D211–D215. doi:10.1093/nar/gkn785. 18940856 PMC2686546

[7] Marchler-Bauer A, Lu S, Anderson JB, Chitsaz F, Derbyshire MK, DeWeese-Scott C, Fong JH, Geer LY, Geer RC, Gonzales NR, Gwadz M, Hurwitz DI, Jackson JD, Ke Z, Lanczycki CJ, Lu F, Marchler GH, Mullokandov M, Omelchenko MV, Robertson CL, Song JS, Thanki N, Yamashita RA, Zhang D, Zhang N, Zheng C, Bryant SH 2011 CDD: a Conserved Domain Database for the functional annotation of proteins. Nucleic Acids Res 39:D225–D229. doi:10.1093/nar/gkq1189. 21109532 PMC3013737

[8] Myers EW, Miller W 1988 Optimal alignments in linear space. Comput Appl Biosci 4:11–17. doi:10.1093/bioinformatics/4.1.11. 3382986

[9] Roberts MD, Martin NL, Kropinski AM 2004 The genome and proteome of coliphage T1. Virology 318:245–266. doi:10.1016/j.virol.2003.09.020. 14972552

[10] Brown KL, Sarkis GJ, Wadsworth C, Hatfull GF 1997 Transcriptional silencing by the mycobacteriophage L5 repressor. EMBO J 16:5914–5921. doi:10.1093/emboj/16.19.5914. 9312049 PMC1170222

[11] Xu J, Hendrix RW, Duda RL 2014 Chaperone-protein interactions that mediate assembly of the bacteriophage lambda tail to the correct length. J Mol Biol 426:1004–1018. doi:10.1016/j.jmb.2013.06.040. 23911548 PMC3907469

[12] Schneider-Scherzer E, Auer B, de Groot EJ, Schweiger M 1990 Primary structure of a DNA (N6-adenine)-methyltransferase from *Escherichia coli* virus T1. DNA sequence, genomic organization, and comparative analysis. J Biol Chem 265:6086–6091. 2180941

[13] Wagner EF, Auer B, Schweiger M 1979 Development of *Escherichia coli* virus T1: escape from host restriction. J Virol 29:1229–1231. 376871 10.1128/jvi.29.3.1229-1231.1979 PMC353285

[14] Casjens SR, Gilcrease EB 2009 Determining DNA packaging strategy by analysis of the termini of the chromosomes in tailed-bacteriophage virions. Methods Mol Biol 502:91–111. doi:10.1007/978-1-60327-565-1_7. 19082553 PMC3082370

[15] Penner M, Morad I, Snyder L, Kaufmann G 1995 Phage T4-coded Stp: double-edged effector of coupled DNA and tRNA-restriction systems. J Mol Biol 249:857–868. doi:10.1006/jmbi.1995.0343. 7791212

[16] Xu M, Struck DK, Deaton J, Wang IN, Young R 2004 A signal-arrest-release sequence mediates export and control of the phage P1 endolysin. Proc Natl Acad Sci USA 101:6415–6420. doi:10.1073/pnas.0400957101. 15090650 PMC404059

[17] Sun Q, Kuty GF, Arockiasamy A, Xu M, Young R, Sacchettini JC 2009 Regulation of a muralytic enzyme by dynamic membrane topology. Nat Struct Mol Biol 16:1192–1194. doi:10.1038/nsmb.1681. 19881499 PMC3075974

[18] Summer EJ, Berry J, Tran TA, Niu L, Struck DK, Young R 2007 Rz/Rz1 lysis gene equivalents in phages of Gram-negative hosts. J Mol Biol 373:1098–1112. doi:10.1016/j.jmb.2007.08.045. 17900620

